# Non-Invasive Topical Drug-Delivery System Using Hyaluronate Nanogels Crosslinked via Click Chemistry

**DOI:** 10.3390/ma14061504

**Published:** 2021-03-18

**Authors:** Hyunsik Choi, Mina Kwon, Hye Eun Choi, Sei Kwang Hahn, Ki Su Kim

**Affiliations:** 1 Department of Materials Science and Engineering, Pohang University of Science and Technology (POSTECH), 77-Cheongam-ro, Nam-gu, Pohang, Gyeongbuk 790-784, Korea; chlgustlr67@postech.ac.kr; 2 School of Chemical Engineering, College of Engineering, Pusan National University, 2 Busandaehak-ro 63 beon-gil, Geumjeong-gu, Busan 46241, Korea; kmn1126@naver.com (M.K.); hehe1722344@gmail.com (H.E.C.); 3 Center for Nanomedicine, Brigham and Women’s Hospital and Harvard Medical School, 45 Francis Street, Boston, MA 02115, USA

**Keywords:** hyaluronate, click chemistry, nanogel, noninvasive, drug delivery

## Abstract

Hyaluronate (HA) has been widely investigated for noninvasive topical drug delivery of chemical drugs and biopharmaceuticals. However, previous noninvasive delivery systems have been facilitated mostly by chemical conjugation of drugs with HA, which can cause reduced therapeutic efficacy and safety issues in chemically modified drugs. Here, HA nanogels were synthesized by crosslinking via “click” chemistry for noninvasive topical delivery of a model drug without chemical modification. The model-drug-encapsulating HA nanogels could be uptaken to the skin melanoma cells in vitro by HA-mediated endocytosis. In addition, histological analysis showed that HA nanogels could be topically delivered to the deep skin and tongue tissues through the noninvasive delivery routes. Taken together, HA nanogels could be effectively used for the noninvasive topical delivery of various therapeutic drugs.

## 1. Introduction

A variety of noninvasive drug-delivery systems, such as oral, pulmonary, ocular, nasal and transdermal delivery, have been presented as attractive alternatives to needle-based drug delivery with great patient compliance [1,2,3,4,5]. Among them, microneedles have been considered as a promising delivery platform due to their fast onset of action, less invasive manner, better patient compliance and improved permeability [6]. Nevertheless, they have some technical issues, including the possibility of skin irritation or allergy in sensitive skin, and safety issues such as microneedle tips remaining inside the skin [7,8]. Needle-free topical delivery without involving physical damage to the natural skin barrier is greatly advantageous, as it can further resolve compliance issues, such as needle-induced anxiety, and is convenient for drug delivery. However, this long-standing goal has continued to exist, since the skin barrier prohibits the penetration of most large and hydrophilic drug molecules, such as proteins, peptides and nucleotides [9,10,11].

As a noninvasive delivery carrier, hyaluronate (HA) has been widely investigated due to its outstanding features. HA is a natural linear polysaccharide composed of disaccharide units of D-glucuronic acid and N-acetyl-D-glucosamine. Since HA is biodegradable, biocompatible and nontoxic in various biological functions, it has been considered as one of the best candidates as a drug-delivery carrier. The hygroscopic property of HA can hydrate the stratum corneum, regarded as a skin barrier, and the hydrophobic patch domain on the backbone of HA can facilitate penetration through the skin. In addition, HA receptors distributed in keratinocytes and fibroblasts may facilitate the localization of HA in skin tissue [12,13,14,15]. Remarkably, HA has been shown to effectively deliver not only drugs, but also nanoparticles to the deep skin tissue, with high efficiency [16,17,18,19,20]. Previously, Kim et al. [16] carried out noninvasive transdermal delivery of vaccines (ovalbumin, OVA) using HA as a drug carrier, and confirmed HA-OVA conjugates could penetrate into the skin and deliver drugs to the deep skin tissue even in the case of high-molecular-weight protein drugs In addition, Yang et al. [17] reported an HA–human growth hormone conjugate for transdermal delivery. With enhanced penetration into the skin, the HA conjugate showed a proliferative effect on keratinocyte and fibroblast, with an elevated expression level of phosphorylated Janus kinase 2 (p-JAK2). However, since this delivery requires chemical conjugation between the drug and carrier molecules, the drugs, especially biopharmaceuticals, can be denatured, and their therapeutic efficacy reduced by the chemical modification.

In this work, we developed a topical-delivery nanoplatform based on HA nanogels encapsulating a model drug to circumvent the above issues. We used the “click” chemistry reactions between methyltetrazine (Tz) and *trans*-cyclooctene (TCO) for in situ formation of HA nanogels. The click reaction between Tz and TCO is known to occur with second-order rate constants of up to 3.3 × 10^6^/M·s, which shows high selectivity at low concentrations and high biocompatibility [21]. Accordingly, this chemistry is suitable for in situ crosslinking of polymers, and thus has been widely applied in biomedical applications [22,23]. Furthermore, since nanogels have a porous structure, drugs can be physically encapsulated in the pore of nanogels without further chemical reaction. Therefore, we assessed the feasibility of HA nanogels as a noninvasive topical drug-delivery carrier in vitro and in vivo.

## 2. Materials and Methods

### 2.1. Materials

Sodium hyaluronate (HA) with a molecular weight (MW) of 100 kDa was purchased from Lifecore Co. (Chaska, MN, USA). 1-Ethyl-3-(3-dimethylaminopropyl) carbodiimide (EDC), N-hydroxysulfosuccinimide (sulfo-NHS), diaminohexane (DAH) and dimethyl sulfoxide (DMSO) were purchased from Sigma Aldrich (St. Louis, MO, USA). Methyltetrazine (Tz)-PEG-NH_2_ and *trans*-cyclooctene (TCO)–PEG–NH_2_ were purchased from Click Chemistry Tools (Scottsdale, AZ, USA). Dulbecco’s modified Eagle’s medium (DMEM), fetal bovine serum (FBS), penicillin and phosphate-buffered saline (PBS) were purchased from Invitrogen Co. (Carlsbad, CA, USA). Cyanine 5.5 (Cy5.5) and fluorescein isothiocyanate (FITC) dye were obtained from Lumiprobe Co. (Hunt Valley, MD, USA).

### 2.2. Synthesis of HA–TCO and HA–Tz Conjugates

HA–Tz and HA–TCO conjugates were prepared using simple EDC/NHS chemistry between the amine group of Tz–PEG and TCO–PEG, and the carboxyl group of HA. Briefly, 30 mg of HA was dissolved in 4 mL of Deionized (DI) water, and each click molecule (10 M ratio of HA repeating unit) was dissolved in 100 μL of DI water. Then, the solution of click molecules was added to the HA solution in a dropwise manner. After reaction overnight with EDC and sulfo-NHS as catalysts, the resulting solution was dialyzed against a large excess amount of water using a prewashed dialysis membrane tube (MWCO of 10 kDA) and lyophilized for 3 days. The HA-click conjugates were characterized by ^1^H NMR in D_2_O. The degree of substitution was calculated by the following equation:

(1) $$\mathrm{Degree}\;\mathrm{of}\;\mathrm{substitution}\;(\%)\;=\;\frac{I_{C}\;\times \;N_{HA}}{N_{C}\times \;I_{HA}}\;\times \;100$$

where *I_C_* and *N_C_* are an integral and number of characteristic peak of click molecules (δ = 7.0–7.2 ppm for *Tz* and δ = 5.5–6.0 ppm for TCO), respectively, and *I_HA_* and *N_HA_* are those of protons in the acetamido moiety of HA (at δ = 2 ppm).

### 2.3. Preparation of HA Nanogels

The same number of HA–Tz and HA–TCO conjugates were dissolved in DI water. Then, these solutions were mixed slowly during sonication with a probe ultrasonicator (VCX-750 Vibra cell, Sonics, Newtown, CT, USA). After sonication for 30 min, the resulting solution was filtered with a syringe filter (pore size of 220 nm). The size and zeta potential of the HA nanogels were characterized by dynamic light scattering (DLS, Zetasizer Nano-ZS, Malvern, UK) and a cryotransmission electron microscope (Cryo-TEM, Tecani Polara F30, Thermo Scientific, Waltham, MA, USA). To investigate further, FITC dye was encapsulated in the HA nanogels with the same procedure, or HA nanogels were labeled by chemical conjugation of Cy5.5 dye.

### 2.4. In Vitro Cell-Uptake Experiment

The mouse melanoma B16F1 cell line was obtained from American Type Culture Collection (ATCC, Manassas, VA, USA) and the cells were cultured on 96-well plates at a density of 10^4^/mL. FITC-encapsulating HA nanogels were incubated with cells in serum-free medium supplemented with 10% FBS, 100 IU/mL penicillin and 100 mg/mL streptomycin at 37 °C in a humidified incubator containing 5% atmospheric CO_2_. To investigate the cellular uptake of nanogels, the cells were incubated with FITC as model-drug-encapsulating nanogels (2 mg/mL) for predetermined time points (1 and 2 h). In addition, to confirm HA receptor-mediated endocytosis of the HA nanogels, a competitive binding test was conducted in the presence of excess HA at a concentration of 20 mg/mL for 2 h preincubation. After washing with PBS, the B16F1 cells were fixed in 4% paraformaldehyde solution at room temperature for 20 min. Then, the cells were mounted with Vectashield mounting (Seven Hills, Australia) medium containing 4,6-diamidino-2-2phenylindole (DAPI). Fluorescence images were obtained by confocal microscopy with a Leica CM 1850 cryostat (Leica, Deerfield, IL, USA).

### 2.5. In Vivo Noninvasive Delivery Experiment

The 8-week-old wild-type BALB/c mice bred in pathogen-free facilities at Harvard Medical School (HMS) were used in this study. HA nanogel labeled by conjugation with Cy5.5 dye (50 μg) was topically applied on the hair-removed mouse skin and sublingual region. After 4 h, the retrieved skin and tongue tissues were fixed in 4% paraformaldehyde solution, embedded into optimal cutting temperature compound (OCT) at −70 °C and cut into 5 μm-thick sections. The sections were fixed with cold acetone at −20 °C and washed with distilled water to remove the residual OCT resins on the slide. Histological tissue sections were imaged by using a confocal microscope. The image analysis was performed using Image J (NIH, Bethesda, MD, USA). All live-animal experiments were approved by the HMS Institutional Animal Care and Use Committee (#04327, #05052).

## 3. Results and Discussion

### 3.1. Preparation and Characterization of the HA Nanogel

**Figure 1:**
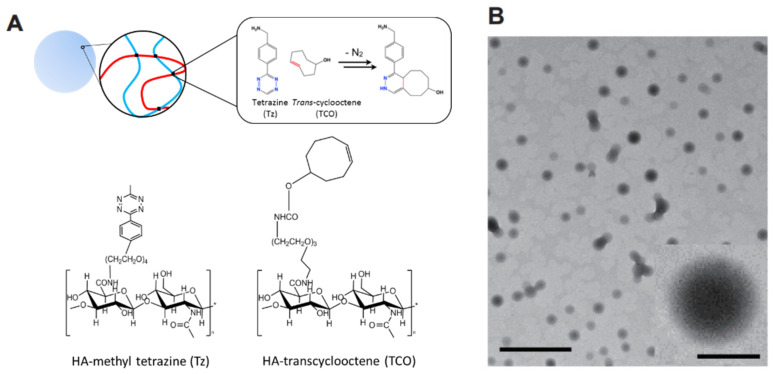
(**A**) Schematic illustration of HA nanogels crosslinked via click chemistry between methyl tetrazine (Tz) and *trans*-cyclooctene (TCO). (**B**) The Cryo-TEM image of HA nanogel (scale bar = 500 nm). Inset shows the expanded image of an individual nanogel particle (scale bar = 100 nm).

**Figure 2:**
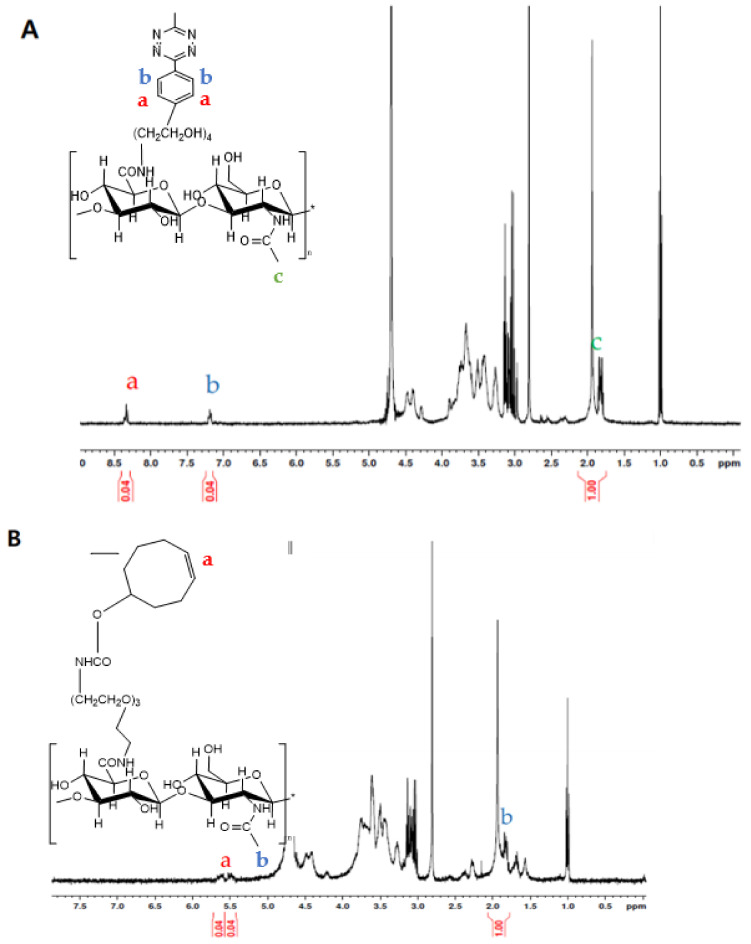
^1^H NMR spectra of (**A**) HA–Tz conjugates and (**B**) HA–TCO conjugates in D_2_O. Inset shows the molecular structure of each conjugate and the position of characteristic proton peaks.

The HA nanogels were synthesized by bioorthogonal reaction of Cu-free click chemistry between Tz and TCO. As schematically shown in Figure 1, the HA–Tz and HA–TCO conjugates were crosslinked without any catalyst and formed hydrogel nanoparticles (nanogels). Each conjugate was synthesized using simple EDC/NHS chemistry between the amine group of Tz or TCO and the carboxyl group of HA. According to ^1^H NMR analysis (Figure 2A,B), since the characteristic peaks corresponding to both HA and TCO or Tz appeared in the NMR spectra, we could confirm the successful preparation of the HA–Tz and HA–TCO conjugates. The peak at δ = 1.95–2.0 corresponded to the acetamido moiety of HA (Figure 2). In addition, the peaks at δ = 7.0–7.2 ppm and δ = 8.0–8.2 ppm corresponded to the Tz (Figure 2A), and the peak at δ = 5.5–6.0 ppm was related to the grafted TCO (Figure 2B). The degree of substitution was 6% for HA–Tz was 6% and 12% for HA–TCO.

Using the conjugates, HA nanogels were formed by click-chemistry crosslinking between Tz and TCO of each aqueous solution. During gelation, probe ultrasonicator broke up the gel to form a HA nanogels. The nanogels were well-dispersed in water. Cryo-transmission electron microscopy (Cryo-TEM) revealed the well-dispersed and spherical shaped HA nanogels with a size of ca. 180 ± 7 nm (Figure 1B) and the surface charge of nanogels was measured to −23.5 mV by Zetasizer.

### 3.2. In Vitro Drug Release

**Figure 3:**
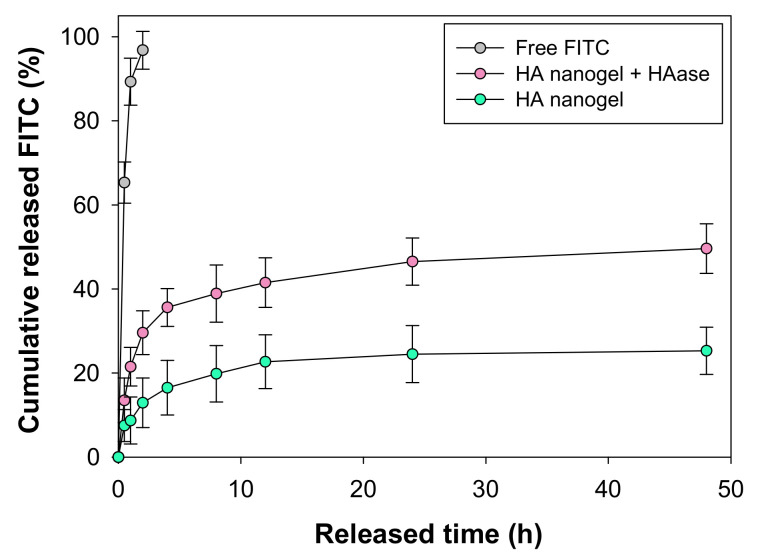
In vitro release test of FITC-encapsulating HA nanogel for 48 h.

In order to confirm the feasibility of HA nanogels as a noninvasive drug delivery carrier, we investigated in vitro release profile of a fluorescent model drug of fluorescein isothiocyanate (FITC) for 48 h by UV-vis spectroscopy (Figure 3). The encapsulating efficiency of FITC was 24 ± 5%. This is because TCO and Tz create sufficient hydrophobicity in the HA nanogel to allow FITC loading via hydrophobic and π-π stacking interactions, indicating that several drugs containing aromatic rings can be encapsulated in the HA nanogels. As shown in Figure 3, encapsulated FITC was slowly released to 25.3% for 48 h. Meanwhile, Free FITC was explosively released to 100% within 2 h. Moreover, after the addition of hyaluronidase into the nanogel solution, HA was degraded and FITC release was increased to 49.6%, indicating that FITC was stably encapsulated in the HA nanogel.

### 3.3. In Vitro Cellular Uptake of HA Nanogels

**Figure 4:**
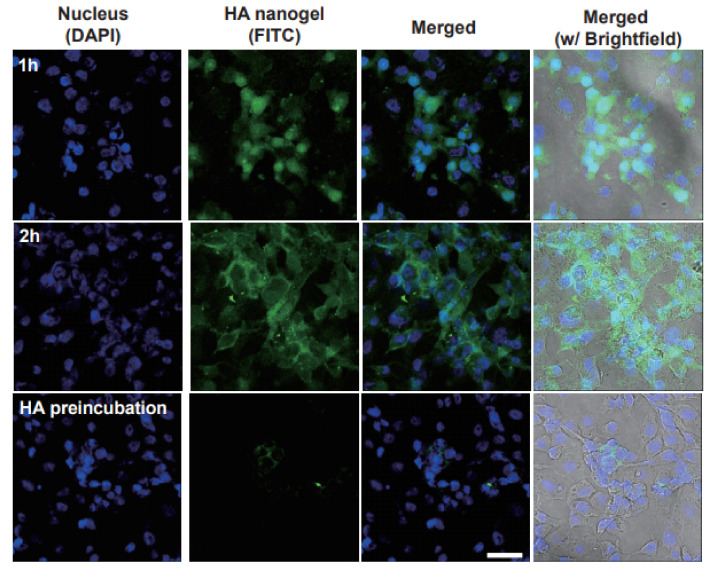
In vitro confocal microscopic analysis of FITC-encapsulating HA nanogels uptaken to B16F1 cells after 1 h (**top**) and 2 h (**middle**) of incubation, and the competitive binding test with HA preincubation (**bottom**). Scale bar indicates 50 µm.

To investigate in vitro cellular uptake of the HA nanogel carriers, FITC as a fluorescence indicator was encapsulated in the HA nanogels. Due to a negative charge, HA nanogels were uptaken into the B16F1 cells by the HA receptor-mediated endocytosis. We performed the incubation of the HA nanogels containing FITC with B16F1 cells, which are known to express HA receptors such as CD44 and LYVE-1 [24,25,26,27]. After incubation for a predetermined period (1 h, 2 h), confocal microscopic analysis was carried out (Figure 4). The in vitro bioimaging using HA nanogels showed the target-specific intracellular delivery of HA derivatives to the B16F1 cells with HA receptors. After incubation of the HA nanogels for 1 h, we could confirm the efficient uptake of nanogels into the melanoma cells. Furthermore, the amount of uptaken nanogels into the B16F1 cells was increased with increasing time (top and middle rows in Figure 4). In addition, we investigated the competitive cellular uptake of the HA nanogels with preincubation of free HA to confirm the HA receptor-mediated endocytosis. As shown in Figure 4, the preincubation of intact HA appeared to reduce the cellular uptake of HA nanogels drastically. The results of the competitive binding test with preincubation of free HA clearly supported the HA receptor-mediated endocytosis of the HA nanogels.

### 3.4. In Vivo Noninvasive Topical Delivery of HA Nanogels

**Figure 5:**
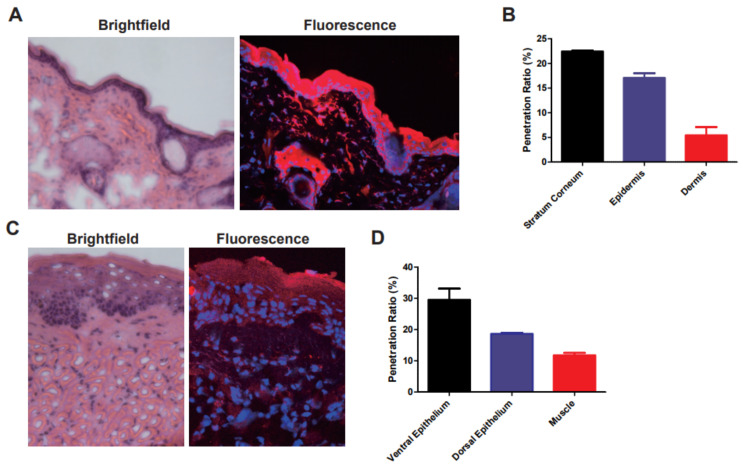
(**A**) The histological analysis (H&E staining, left) and fluorescence image (right) of skin acquired by confocal microscopy after topical application of Cy5.5-labeled HA nanogels, and (**B**) the penetration ratio of Cy5.5-labeled HA nanogels in each layer of skin. (**C**) The histological analysis and (**D**) penetration ratio for underside of tongue.

On the basis of the results for cellular uptake, we assessed in vivo penetration of the nanogels into mice skin and sublingual region. The HA nanogels labeled with Cy5.5 were topically applied on mice back skin and sublingual region. In our previous work, we could confirm that HA–ovalbumin (OVA) conjugates had successfully penetrated into the skin barrier (stratum corneum) and were well dispersed in the epidermis and dermis layers [16]. In addition, HA–poly(lactide-co-glycolide) (PLGA) nanoparticles encapsulating minoxidil also showed successful transdermal penetration [28]. However, the other groups without HA conjugation could not be delivered to the deep skin tissues. In accordance with previous results, HA nanogels could be delivered and well dispersed in deep skin tissues, possibly by the HA receptor-mediated endocytosis. Confocal microscopic images of tissue sections harvested at 4 h post-topical administration showed significant penetration of HA nanogels into the skin (Figure 5A). For the quantitative analysis, we measured the spatial distribution of Cy5.5 fluorescence. At 4 h, the remaining solution outside the skin was collected and analyzed, and showed that 55% of the total applied Cy5.5 labeled to the HA nanogels remained in the solution. Assuming that the rest of agents had penetrated into the tissues, 23% of the total nanogels appeared to be present in the stratum corneum, 17% in the epidermis and 5% in the dermis (Figure 5B). Of the total 45% of HA nanogels that were absorbed in the skin, about 49% (= 22/45) of those were delivered to the epidermis and dermis. In addition, the HA nanogels also exhibited excellent penetrative behaviors in the sublingual region, as shown in the histological analysis (Figure 5C). The 40% of the total Cy5.5 labeled in HA nanogels remained in the solution. Assuming that the rest of agents had penetrated in the tissues, 30% of the total nanogels were present in the ventral epithelium, 19% in the dorsal epithelium and 11% in the muscle (Figure 5B). About 50% (=30/60) of them were delivered to the deep tongue.

Notably, compared to our previous work [16,28] on transdermal vaccine delivery, the HA nanogel carriers showed high efficiency of tissue penetration, possibly due to their soft and flexible characteristics. In case of the HA–OVA conjugates, the efficiency of tissue penetration was only 39% due to the steric effect of large-size OVA (protein), and 41% of minoxidil (chemical drug) could penetrate with rigid HA–PLGA nanoparticles. Although the nanogels had a relatively large size, their flexible structure and softness might facilitate penetration into tissue barriers. In addition, in the case of tongue, the structure of ventral epithelium is looser than the dense stratum corneum. Because of the density of cells on the top layers, more HA nanogels might be penetrated into the deep tissue. In addition, there was no inflammation observed in brightfield microscopic images after hematoxylin and eosin (H&E) staining of both tissues.

HA nanogels might penetrate into deep skin and tongue, where blood vessels are located, and degraded by hyaluronidase after binding to HA receptors on the cell membrane. Then, drugs might be delivered to the blood vessels and systemically circulated into the body. Taken together, the HA nanogel platform could be successfully harnessed to develop novel noninvasive topical drug delivery system via various routes with high delivery efficiency and patient compliance.

## 4. Conclusions

HA nanogels crosslinked via click chemistry were successfully developed for noninvasive topical drug delivery. HA–Tz and HA–TCO conjugates were synthesized and mixed together to prepare HA nanogels. In vitro and in vivo tests showed that the HA nanogels were able to encapsulate drugs stably and deliver the drugs noninvasively and reliably through transdermal and sublingual routes. This HA nanogel system is expected to be effectively utilized for noninvasive topical drug delivery via various routes.

## Data Availability

The data presented in this study are available on request from the corresponding author.
